# Supplementary material for: Drivers of Lassa fever in an endemic area of southwestern Nigeria (2017–2021): An epidemiological study
Source: PLoS One. 2025 Jun 25;20(6):e0327143. doi: 10.1371/journal.pone.0327143 (PMC12193570; doi:10.1371/journal.pone.0327143)
Supplement: Attachment — Submitted filename: Response to Reviewers Comments (2).docx [file pone.0327143.s002.docx]

**RESPONSE TO REVIEWER’S COMMENTS**

Reviewer #2: The methodology mentioned in the provided text seems suitable for conducting a cross-sectional study using secondary data from the Integrated Disease Surveillance and Response (IDSR) weekly epidemiological data line list for a specific time period. However, here are a few points that can be improved or clarified in the methodology:
1. Sample selection: The text does not mention how the sample of suspected cases was selected or if all suspected cases in the given time period were included in the analysis. Clarifying the inclusion criteria and sampling method would provide more transparency.

***Response: Samples of suspected cases were selected based on the definition provided in the manuscript, see lines 177-183, and all the suspected cases identified, in the data under review, were included in the analysis.***

2. Data quality: The text does not explicitly mention the quality of the secondary data used. It would be helpful to discuss any processes undertaken to ensure the reliability and accuracy of the data, such as data validation or data cleaning procedures.

***Response: Information on data quality and other issues raised have been included in the “methods section” (under Data source/quality). See lines 161-167.***

3. Data used in the study is claimed to be of five year data yet, It includes a date range of approximately four years from 2017 to 2021, without specifying the exact months of commencement and conclusion.

***Response: The specific period of the year in the data considered for this study has been stated in the methods section. See lines 137, 145, 157.***

Reviewer #2: So far, the results and data analysis method appear to be correct and aligned with the objectives. However, they only require a proper explanation in proper English language.

***Response: Proper review of the results and data analysis method has been carried out..***

Reviewer #2: The conclusion section has several issues. Instead of providing a brief summary of the major findings, it immediately jumps to recommendations. The recommendations include multiple intervention options, which are numerous and some of them are not directly related to the study. The study was based on secondary data, which only provides insights into the occurrence and survivability of infected individuals. It cannot identify actual transmission patterns and major factors contributing to disease outbreaks, making it inappropriate to make recommendations based on these data.

Overall, the weakness lies in the lack of specific details, feasibility assessment, and contextual analysis. Without addressing these weaknesses, it is difficult to determine the effectiveness and practicality of the proposed solutions for mitigating LF.

***Response: The authors appreciate the reviewer for this observation. However, the authors wish to state that the recommendations were made based on the findings of the study. The interventions mentioned in the recommendations are suggested proposed steps that could mitigate LF cases in the study area. Overall, this section has been streamlined and summarised to provide more focused recommendations***
