# Supplementary material for: Drivers of Lassa fever in an endemic area of southwestern Nigeria (2017–2021): An epidemiological study
Source: PLoS One. 2025 Jun 25;20(6):e0327143. doi: 10.1371/journal.pone.0327143 (PMC12193570; doi:10.1371/journal.pone.0327143)
Supplement: Attachment — Submitted filename: 2-Response to Reviewers Comments_PONE-D-24-05209.docx [file pone.0327143.s004.docx]

Reviewers' comments:

Reviewer's Responses to Questions

**Comments to the Author**

5. Review Comments to the Author
Reviewer #1: The authors make a strong case for using pre-existing public health data to describe the demographics of an endemic viral infection, Lassa Fever, as well as the role of poor biosafety practices in conjunction with a known rodent vector, resulting in a high disease burden. The authors’ analysis of state-level data provided by local government areas in Nigeria clearly depicts the high disease burden incurred by local populations, its significant (~25%) case-fatality rate, and highlights those patients at greatest risk.

A few suggested edits:

(lines 365-66) “Our findings showed that confirmed LF cases were mostly (28.7%) adults aged 45 years old and above.”

***Response: The sentence has been edited as:***

***“Findings from this study revealed that majority (28.7%) of the LF cases were adults aged 45 years and above”. Check lines 364-365.***

This conclusion is confusing and does not appear to be supported by the included data from Table 2. Per this table, ~50% of confirmed cases are adults under 45. While case-fatality rates show the greatest mortality in patients >40 with confirmed LF, the substantial majority of confirmed LF patients are <45. This point should be clarified.

***Response: Table 2 has been thoroughly reviewed, to capture the statement in the conclusion.***

(lines 372-73) “Our study did not explore co-incident health conditions in the population studied.

”The authors suggest that increased mortality in the older cohort may stem from increased comorbid conditions, while also admitting that no analysis of these “co-incident health conditions” is included in the study. Again, although these conclusions may be intuitively correct, the authors present no data to support them.

***Response: Thank you for this comment. The authors are not making any categorical assertion here. As the reviewer rightly pointed out, underlining health conditions is a common occurrence as individuals get older. It is against this backdrop that the authors associate likelihood of increased CFR to older individuals.***

Similarly, in the abstract, Results and Discussion sections, the authors mention open air markets. sun drying of food, and congested urban areas with significant rodent presence as possible contributing factors to the increased incidence of LF within these populations, but no evidence is provided to support this contention. The authors refer to key stakeholder interviews and environmental assessments but no references or supporting documentation are included for these resources.

***Response: The authors appreciate the reviewer for this comment The State DSNO provided information regarding possible risk practices or activities driving LF outbreaks. Additionally, other hazards and vulnerabilities noticed through the observation of the environment around the neighbourhood and major market settings were reported. None of these activities involved a standard questionnaire administration or checklist assessment, thus there is no formal document to present. However, a statement has been added for better clarity. Check line 226/ 227.***

Secondary data use in public health surveillance is inherently limited as specific data that might be desired by the study authors may be absent or insufficiently detailed to be of use in subsequent analyses. An additional discussion of these limitations may be beneficial.

***Response: Additional statement has been included. Check line 395-397***

(lines 401-402) “integrate agricultural activities with health education to mitigate LF at local levels”
This leading conclusion from the study appears to be a recommendation that embraces a common-sense approach to infectious disease mitigation, although again, the rationale underlying this policy change is not supported by evidence.

***Response: The authors agree with the reviewer. This conclusion was inferred from the findings of the study, and it was intended to serve as a recommendation that will help in the mitigation of the present challenge of LF.***

Discussion of One Health strategies for LF mitigation and control may benefit from a sentence describing the WHO’s One Health concept and its role in implementing local public health solutions.

***Response: Thank you. A statement on the WHO’s One Health concept, and its role in implementing local public health solutions has been added. Check lines 408-415.***

While improved biosafety practices in this limited-resource environment appears to be a logical long-term goal, the question that arises is this: Is it possible to minimize human-rodent interactions in this socioeconomic sector? While the authors point out that their study emphasized demographic factors and “did not explore other factors such as socioeconomic status, access to healthcare, and environmental conditions,” the solutions offered in the Conclusion section primarily focus on these latter factors.

In summary, the authors provide a clear picture of the demographics of patients with confirmed Lassa Fever infection in specific areas of southwestern Nigeria. Their conclusions on those most at risk for increased LF mortality are well-supported by their secondary data analysis, with a few exceptions noted above. Their public health recommendations, present throughout the manuscript and most notably in the conclusions are reasonable and likely evidence-based, however, the absence of this evidence may undermine the strength of their conclusions. These suggestions may also distract from the primary purpose of the study, a clear, concise description of the demographics of LF infection in a region where LF is an ongoing public health risk.

***Response: Thank you for the observation. Necessary adjustments have been made. Check line 226 & 227.***

Reviewer #2: 1) Make the title concise like "Drivers of Lassa Fever in Southwestern Nigeria (2017-2021): An Epidemiological Study."

***Response: The title has been changed as suggested.***

2) In abstract clarify the methodologies used, particularly the type of regression analysis, to provide a clearer picture to the reader. Additionally, include specific statistical results and p-values in to strengthen the abstract.

***Response:*** ***This section of the manuscript has been edited as shown below:***

***“Kaplan-Meier estimate was used to describe the probability of survival among the LF cases. Also, a univariable binary logistic regression was used to explore factors associated with mortality among the study participants”. Check lines 44-46.***

3) Expand introduction section by adding brief details of Lassa virus transmission and environmental concerns and elaborate One health strategies to minimize case occurrence using suggested articles:
<http://pvj.com.pk/pdf-files/24-182.pdf>
<http://www.pvj.com.pk/pdf-files/23-554.pdf>
<https://scholar.google.com/citations?view_op=view_citation&hl=en&user=RW86akAAAAAJ&citation_for_view=RW86akAAAAAJ:qxL8FJ1GzNcC>
<https://www.researchgate.net/profile/Ghulam-Murtaza-77/publication/378403386_Vector-Borne_Zoonotic_Diseases/links/65d88d0aadf2362b6352da06/Vector-Borne-Zoonotic-Diseases.pdf>

***Response: Thank you. Information from the suggested references has been included in the introduction section. Check lines 124-139.***

4) Introduction could be more focused on identifying the research gap and justifying the study.

***Response: Thank you. Suggested comments have been addressed.***

5) Discuss how the geographic features of Ondo State might influence the spread of Lassa fever to make the epidemiological significance clearer in Geographic Location.

***Response: Thank you. More details have been provided to capture the association of geographical features of Ondo State and the spread of LF. Check lines 169-173.***

6) Address any limitations or potential biases in the data collection process, such as missing data or inconsistencies in reporting, and how they were managed in data source/quality.

***Response: Relevant information has been included. Check lines 212-222.***

7) Data analysis section needs a detailed explanation, provide more detail on the regression models, including the rationale for their selection and any assumptions made. Clarify if any corrections for multiple comparisons were applied.

***Response: This section was revised as below:***

***“A binary logistic regression model was employed to investigate the factors associated with mortality among the LF cases. This outcome was dichotomized as coded as zero (Alive) and one (Dead). A univariable logistic regression model was then fitted to examine factors that were independently associated with mortality among the participants”. Check lines 218-221.***

8) In discussion add more referral studies; compare your findings with similar studies from Nigeria or other West African countries. Highlight any novel insights or contradictions with existing research.

***Response: Thank you. More references have been added.***

9) In the discussion 4th paragraph, Lines 374-385 should be rewritten as data is not well organized.

***Response: Thank you. This section has been reviewed as suggested.***

10) The conclusion looks too general; focus on key findings of this study and mention future interventions that can be done.

***Response: Thank you. The conclusion has been reviewed.***

11) Figures are too blurry to understand, improve quality, cite figures in running text.

***Response: These have been corrected, thank you.***

12) Label Graph Y axis, what is it indicating?

***Response: Thank you for the observation. Figures have been well labelled.***

Reviewer #3: 1) Lack of coherence in data presentation

***Response: Data presentation has been thoroughly reviewed.***

2) Rewrite your abstract for further clarification

***Response: Abstract has been reviewed for clarity.***

3) Significance ( P value) is not mentioned in the abstract

***Response: The statement was recast as: “Age is a strong predictor of survival; hospitalized patients >40 years were significantly more likely than younger ones to experience mortality (Odds ratio:2.46; 95% CI=1.67–3.62; p<0.01).***

***The above statement is to indicate that increasing age increases the likelihood of mortality among participants, also providing the p-value***

4) Modify the images and put the references in the given text as well.

***Response: Thank you. All suggestion has been incorporated.***

5) See it again Source: Department of Geography, University of Ibadan; [https://Grid3.org](https://grid3.org/)

***Response: Thank you for this comment. The map was generated from the source provided and the link*** (https://Grid3.org)***.***

6) <https://www.ijvets.com/pdf-files/24-449.pdf>
7) One health initiative ……..<http://www.pvj.com.pk/pdf-files/24-035.pdf>
8) <https://www.ijvets.com/pdf-files/23-328.pdf>
9) <https://journals.lww.com/international-journal-of-surgery/fulltext/2023/04000/Re_emergence_of_the_Lassa_virus_in_Africa__a.45.aspx>
Suggested for reading, understanding and supporting the manuscript
