# Supplementary material for: Drivers of Lassa fever in an endemic area of southwestern Nigeria (2017–2021): An epidemiological study
Source: PLoS One. 2025 Jun 25;20(6):e0327143. doi: 10.1371/journal.pone.0327143 (PMC12193570; doi:10.1371/journal.pone.0327143)
Supplement: Attachment — Submitted filename: Response to Reviewers Comments-3.docx [file pone.0327143.s005.docx]

Reviewer #4: The efforts of the authors to give appropriate response to the earlier reviewers is recognised and commended. However, some areas especially the discussion needs more attention.
The result from the study reads: Of 1,115 confirmed LF cases, 284 died (case fatality rate, CFR, 25.5%).
Line 249
To enrich this discussion, can you compare the CFR in Ondo State with the national CFR? Line 378-379 This will help in assessing the successes in case management and the effectiveness of other programs aimed at mitigating the impact of Lassa fever.

***Response: Thank you for the comment. The issue raised has been duly addressed to enrich the discussion. Check lines 381-386.***

The conclusion of the study did not stem from the data presented in the study.
Authors are advised to work on the discussion and conclusion sections and clearly indicate the ares of recommendation.

***Response: The conclusion has been reviewed as suggested. Recommendation section has been clearly indicated.***
